# Genetic characterization of pediatric B-cell acute lymphoblastic leukemia in Argentina uncovers molecular heterogeneity and novel variants

**DOI:** 10.3389/fphar.2025.1701680

**Published:** 2025-11-17

**Authors:** María Sol Ruiz, Ezequiel Sosa, Daniel Avendaño, Ignacio Gomez Mercado, María Laura Lacreu, Cecilia Riccheri, Virginia Schuttenberg, Luis Aversa, Elba Vazquez, Geraldine Gueron, Javier Cotignola

**Affiliations:** 1 CONICET - Universidad de Buenos Aires, Instituto de Química Biológica de la Facultad de Ciencias Exactas y Naturales (IQUIBICEN), Buenos Aires, Argentina; 2 Universidad de Buenos Aires, Facultad de Ciencias Exactas y Naturales, Departamento de Química Biológica, Laboratorio de Inflamación y Cáncer, Buenos Aires, Argentina; 3 Hospital Nacional Posadas, Servicio de Hemato-Oncología Pediátrica, Buenos Aires, Argentina; 4 Hospital de Niños Sor María Ludovica, Buenos Aires, Argentina; 5 Hospital de Niños Ricardo Gutiérrez, Unidad de Hematología, Ciudad Autónoma de Buenos Aires, Argentina

**Keywords:** acute lymphoblastic leukemia, molecular subtype, transcriptomics, RNA-seq, biomarkers

## Abstract

**Introduction:**

Acute lymphoblastic leukemia is the most common childhood cancer, yet its diagnosis and risk classification remain incomplete in many regions due to limited access to molecular studies.

**Methods:**

We present the first comprehensive genetic and molecular characterization of childhood B-cell Acute lymphoblastic leukemia in Argentina using whole-transcriptome sequencing of diagnostic bone marrow samples from 32 patients enrolled in the international clinical protocol ALLIC-BFM-2009.

**Results:**

By integrating publicly available bioinformatic tools, we achieved molecular subtyping in over 93% of cases, a significant increase from the 31% rate attained through traditional methods. Our analysis revealed a diverse landscape of known and novel genetic alterations, including gene fusions, single nucleotide variants, and gene expression signatures relevant to prognosis and therapy. Importantly, we identified novel single nucleotide variants in DUX4, CSF3R and CREBBP, and fusion transcripts.

**Discussion:**

This study not only reports transcriptional heterogeneity in our Latin American cohort but also supports the implementation of open-source bioinformatic pipelines in resource-limited settings to enhance precision diagnosis and guide personalized treatment.

## Introduction

Acute lymphoblastic leukemia (ALL) is the most common childhood cancer worldwide. In Argentina, ALL accounted for 29.3% of all pediatric cancers diagnosed between 2000 and 2019, with an average of 395 new cases annually and an age-standardized incidence rate of 4.9 per 100,000 individuals (Moreno and Chaplin, 2021).

Advances in chemotherapy regimens and the stratification of patients into risk groups have led to steady improvements in overall and relapse-free survival. However, despite these advancements, 15%–30% of patients still relapse, often facing severe treatment complications and poor overall survival rates (Oskarsson et al., 2018). Further intensification of traditional chemotherapy is unlikely to yield better outcomes due to the associated acute toxicity and an unfavorable toxicity/benefit ratio (Tallen et al., 2010; McNeer et al., 2020). Therefore, understanding the intrinsic and extrinsic factors that contribute to disease development and treatment failure is crucial.

Childhood ALL outcomes vary significantly worldwide, with survival rates exceeding 80% in most high-income countries, but dropping to 20% in low- and middle-income countries (Chatenoud et al., 2010; Allemani et al., 2018; Pui et al., 2018). The Acute Lymphoblastic Leukemia Intercontinental Consortium (ALLIC) includes countries from Europe and Latin America with the aim of developing common protocols in countries with similar access to diagnostic tools and chemotherapeutic agents, and has recently carried out one of the largest clinical protocols in pediatric ALL, the ALLIC-BFM-2009 protocol, which recruited more than 6,000 patients, 38% of which (N = 2,390) were from Argentina. In this protocol, less than 40% of patients had evaluable cytogenetic or genetic results (Campbell et al., 2023), suggesting there are barriers in achieving genetic subtyping in the clinical setting and representing an important challenge to overcome in the future. The three-year survival rates for pediatric ALL in Argentina have plateaued at ∼76% (69.0%, 76.0%, and 76.1% for the periods 2000–2005, 2006–2011, and 2012–2016, respectively) according to the Argentinian Oncopediatric Hospital Registry, highlighting the need for innovative diagnostic and therapeutic approaches to improve survival. Regarding genetic studies of ALL in Argentina, there are two large studies using cytogenetics, FISH, RT-PCR and MLPA to identify frequent highly prognostic genetic and chromosomal alterations. These studies reported the prevalence of frequent chromosomal rearrangements in ALL, and the prognostic value of IKZF1 deletions (Coccé et al., 2015; Felice et al., 2022), but there are no available data on rare and novel subtypes included in our study.

B-cell ALL (B-ALL) is the most frequent type of ALL, with 27 molecular subtypes (including 4 provisional entities) recognized by the International Consensus Classification (Duffield et al., 2023). These molecular subtypes are characterized by specific chromosomal alterations, gene expression profiles, aneuploidies and point mutations (Brady et al., 2022), which are crucial for guiding risk-adapted therapies and precision medicine. However, the ability to identify these subtypes accurately depends on the availability of molecular diagnostic techniques, which vary across medical centers. The increasing complexity of molecular classification presents significant technical, analytical, and economic challenges, particularly in low- and middle-income countries, limiting the widespread implementation of these advanced diagnostic approaches (Jerez et al., 2024). Moreover, there is a lack of large-scale, international validation studies of RNA-seq for molecular subtyping in Latin America.

This study aimed to perform an integrated molecular characterization of childhood B-ALL in a group of patients enrolled under the ALLIC BFM-2009 protocol in Argentina through transcriptome sequencing. We used a combination of tools requiring different computational resources and bioinformatic skills in order to provide a translational framework for molecular diagnosis. Additionally, we conducted *in silico* analyses to further explore the novel genetic variants identified. Our findings provide valuable insights into the genetic and molecular heterogeneity of B-ALL in a cohort of Latin American patients.

## Materials and methods

### Patients and samples

Bone marrow aspirates (1–5 mL) were obtained from newly diagnosed, untreated children and adolescents with ALL enrolled in the Argentine multicenter clinical protocol ALLIC-GATLA 2010. Samples were collected across three hospitals in Argentina: Hospital Nacional Posadas (n = 8), Hospital de Niños Dr. Ricardo Gutierrez (n = 5), and Hospital Sor María Ludovica (n = 19). The clinico-pathological characteristics and disease outcome were evaluated by trained oncohematologists. All procedures involving human participants complied with the ethical guidelines outlined by the Institutional Review Board (IRB) and with the Declaration of Helsinki. The study protocol was also approved by Argentina’s National Drug, Food and Technology Administration (ANMAT).

The inclusion criteria were: 1) IRB protocol approval, 2) *de novo* primary ALL, 3) patient age between 1 and 18 years old, 4) written informed consent from parents or legal guardians, with written informed assent from patients when applicable. Risk stratification was conducted in accordance with the protocol guidelines based on age, white blood cell count at diagnosis, percentage of blasts at day 8, minimal residual disease (MRD) at days 15 and 33 (evaluated by multiparametric flow cytometry), ploidy, t (9; 22) *BCR::ABL1*, and t (4; 11) *KMT2A::AFF1*, as it follows: Standard Risk (age: 1–5 years, WBC < 20 × 10^9^/L, blasts D8 < 1000), Intermediate Risk (age < 1 year or > = 6 years, WBC > = 20 × 10^9^/L, blasts D8 < 1000), High Risk (t (9; 22) or t (4; 11), or blasts D8 > = 1000, or hypodiploidy). Patients were re-classified to higher risk groups according to their Minimal Residual Disease at day 15 and 33 assessed by flow cytometry.

#### Transcriptome sequencing

Frozen cell pellets in Trizol or purified total RNA from prospective samples were shipped to the Inflammation and Cancer Laboratory at Facultad de Ciencias Exactas y Naturales, Universidad de Buenos Aires (CABA, Argentina). Total RNA was isolated using either Trizol (Thermo Fisher Scientific, United States) or Quick-Zol (Kallium technologies, Argentina) following the manufacturer’s protocols, either at the extraction site or in our laboratory. The RNA was resuspended in RNAse/DNase-free distilled water and stored at −80 °C. RNA quantification and integrity were evaluated using a Nanodrop (Thermo Fisher Scientific, United States) and Bioanalyzer (Agilent Technologies, United States).

Transcriptome sequencing was performed on 32 bone marrow aspirates collected at the time of diagnosis, after selecting samples with high RNA quality. RNA-seq libraries were prepared following ribosomal RNA depletion using the Ribo-Zero Plus rRNA Depletion Kit (Illumina, United States). Paired-end RNA sequencing was conducted at Macrogen (Korea) on HiSeq2000 or HiSeq2500 Systems (Illumina, United States) in five separate batches over 3 years. Transcriptome sequencing data is available at the European Nucleotide Archive repository under the Accession PRJEB80172.

#### Bioinformatic methods

RNA-seq quality was assessed using *FastQC* (v0.11.5, RRID:SCR_014583). Reads were pseudoaligned to the GRCh38 reference transcriptome using Kallisto (RRID:SCR_016582) (Bray et al., 2016) to obtain transcript counts. Transcripts were annotated using the EnsDb.Hsapiens.v86 R package (Bioconductor). SNVs and small InDels were evaluated using RNAmut (Gu et al., 2020) based on a custom index of 114 genes and 79 fusion genes associated with ALL (Supplementary Material S1). Variants were annotated and filtered using an in-house pipeline (Supplementary Material S2) with the following criteria: A) number of reads: MutReads > = 15; B) Variant Allele Fraction in RNA: VAF > = 0.2; C) frequency in population databases: Freq_1000genomes <1% or not available; D) clinical significance in ClinVar (RRID:SCR_006169); E) association with leukemia phenotype; and F) pathogenicity predictions from Polyphen (RRID:SCR_013189). Variants identified in the RNA-seq were confirmed by RT-PCR and Sanger sequencing.

Novel fusion genes were identified using STAR-Fusion (v1.11.0, RRID:SCR_025853) (Haas et al., 2019) and FusionInspector (Haas et al., 2023). Both tools were run on Singularity at the CCAD-UNC (Centro de Cómputo de Alto Desempeño, Universidad Nacional de Córdoba). Molecular subtypes were predicted with the ALLsorts (Schmidt et al., 2022) and ALLCatchR (Beder et al., 2023) classifiers on the high-performance cluster (CeCAR) at Facultad de Ciencias Exactas y Naturales, Universidad de Buenos Aires. Protein structural analysis was performed using the VMD software (RRID:SCR_001820, VMD is developed with NIH support by the Theoretical and Computational Biophysics group at the Beckman Institute, University of Illinois at Urbana-Champaign).

### Statistical analysis and graphics

Statistical analyses, tables and graphics were generated using Rstudio with the following packages: *tidyverse*, *gtsummary*, *plotly, survival, survminer, reshape2* and *chimeraviz*. The oncoprint chart was created using the script available in: https://github.com/sarahet/The_Distinct_DNA_Methylome_ALL/blob/main/Figure5_Extended_Data_Figure5.R.

## Results

### Cohort demographics

The Intercontinental Berlin-Frankfurt-Münster Study Group is a cooperative organization that brings together countries from Europe, Latin America and, initially, Asia, that has conducted two large clinical trials in childhood ALL, with a focus on providing support and guidance to countries with limited resources (Ribeiro and Conter, 2023). The patients included in our study were enrolled under the ALLIC BFM-2009 protocol through the Argentine multicentric protocol ALLIC-GATLA (Grupo Argentino de Tratamiento de la Leucemia Aguda) 2010–2020 (Campbell et al., 2023). We recruited 32 patients diagnosed with primary *de novo* B-ALL. Demographic and clinico-pathological data are shown in Table 1. In summary, the median age at diagnosis was 7 years (range: 2–18 years), with a sex distribution of 47% boys and 53% girls. The studied cohort was composed of 9% of patients categorized as standard-risk, 69% as intermediate-risk and 22% as high-risk. Risk group classification was assigned by the treating physicians according to the ALLIC-BFM-2009 guidelines.

**TABLE 1:** Demographic and clinico-pathological data.

Variable	Total n = 32
Age at diagnosis	
median (range) IQR	7.0 (2–18) years 3–13 years
Sex	
Female/male	17/15 (53%/47%)
ALL subtype	
common/pre-B	26 (81%)/6 (19%)
Risk group (ALLIC-BFM-2009)	
standard/intermediate/high	3 (9.4%)/22 (68.7%)/7 (21.9%)
Blast count in bone marrow	
median (range), IQR	90 (25%–100%), 83%–98%
Response to prednisone, day 8	
good (<1.0 × 10^9^/L)/poor (≥1.0 × 10^9^/L) blasts in blood	28 (88%)/4 (13%)
Minimal residual disease, day 15	
<0.1%/0.1%–10%/>10%/unknown	11 (37%)/14 (47%)/5 (17%)/2
Acute severe toxicity related to treatment	
Yes/No/unknown	9 (29%)/22 (68%)/1 (3%)
Relapse	
Yes	5 (16%)
Death	
Yes	5 (16%)
Follow-up time (months)	
median (range) IQR	32 (2–52) months 25–35 months

IQR, interquartile range.

#### Molecular subtyping using RNA-seq-based classifiers

**FIGURE 1:**
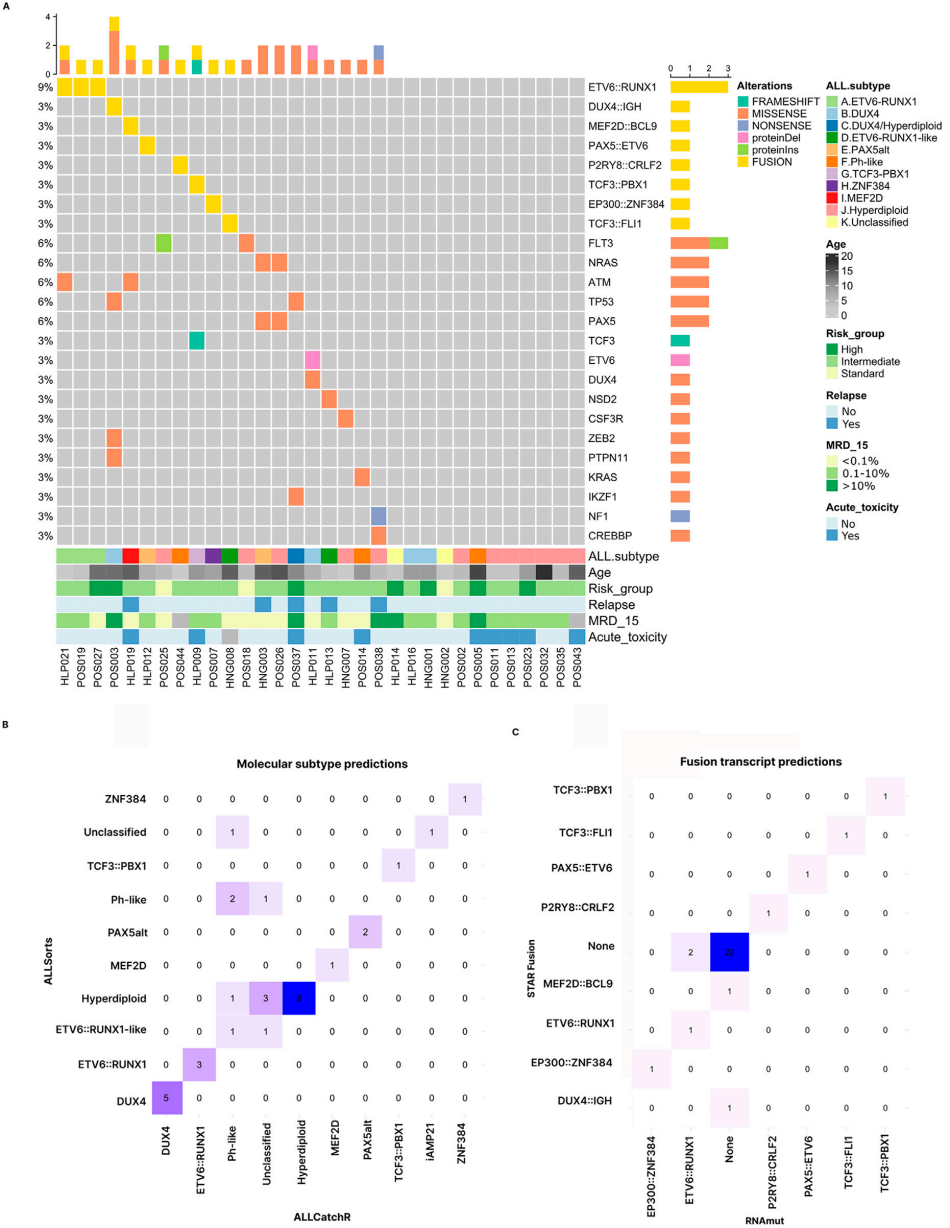
Summary of molecular alterations identified in this study and agreement of methods for subtype and fusion transcript identification. **(A)** Overview of the genomic landscape across the cohort, where each column represents an individual patient and each row corresponds to a specific genetic alteration. SNVs, InDels, and fusion transcripts are displayed, ordered by their frequency within the cohort. ProteinDel: deletions; proteinIns: insertions. Molecular subtypes and clinical features are shown below chart. **(B)** Confusion matrix comparing molecular subtyping results from ALLSorts and ALLCatchR. **(C)** Confusion matrix comparing the identification of fusion transcripts by STAR-Fusion and RNAmut. Only fusion transcripts previously reported in ALL were included in these analyses.

We used the ALLSorts classifier, a machine learning algorithm designed to categorize B-ALL patients into 18 predefined molecular subtypes based on whole transcriptome sequencing data (Schmidt et al., 2022). Since ALLSorts indicates that misclassification often occurs with the “high hyperdiploid”, “low hyperdiploid”, and “near haploid” subtypes, and classification accuracy improves when using the meta-subtype “High Sig”, we opted to use this meta-subtype and called it “hyperdiploid”. ALLSorts successfully assigned the molecular subtype to 30 out of the 32 patients (93.7%); the remaining 2 were categorized as B-other/unclassified (Figure 1A; Table 2). In the case where cytogenetic and molecular test results were available, the classification showed high concordance (17/18 samples, 94%). Of note, the 2010–2020 guidelines for childhood ALL subtyping required testing for only the five most frequent and validated prognostic subgroups (*ETV6::RUNX1*, *BCR::ABL1* (p190 and p210), *KMT2A::AFF1*, hyperdiploid and hypodiploid), limiting the confirmation of the 13 additional subtypes assigned by ALLSorts. We observed one discordant result, a sample that was classified as DUX4 by ALLSorts and as hyperdiploid by traditional karyotyping (POS037, Figures 1, 4). In summary, we identified 9 B-ALL subtypes in our cohort, with hyperdiploid (37.5%), DUX4 (12.5%) and ETV6::RUNX1 (9.4%) being the most frequent (Figure 1; Table 2). In addition, the use of RNA-seq followed by ALLSorts increased the molecular subtyping rate from 31.2% (limited by the mandatory molecular and genetic tests in the ALLIC-GATLA-2010 protocol) to 93.7% (Table 2). It is important to note that the small sample size of this study limits generalization of observed frequencies and they remain descriptive until further validation in a larger cohort.

**TABLE 2:** Summary of molecular subtypes identified in this study and their comparison with ALLIC-BFM-2009 frequencies (Campbell et al., 2023).

	Study			
	GATLA-2010 n = 32			ALLIC-BFM-2009 n = 6,187
B-ALL subtype	GATLA	ALLSorts	Final classification	
B-other/unclassified	12 (37.5%)	2 (6.3%)	2 (6.3%)	582 (9.4%)
BCL2/MYC	N/A	0 (0%)	0 (0%)	N/A
DUX4	N/A	5 (15.6%)	4 (13%)^a^	N/A
Hyperdiploid	8 (25%)	12 (38%)	12 (38%)^a^	762 (12.3%)
DUX4/Hyperdiploid	na	na	1 (3.1%)^a^	na
ETV6::RUNX1	2 (6.2%)	3 (9.4%)	3 (9.4%)	845 (13.7%)
ETV6::RUNX1-like	N/A	2 (6.3%)	2 (6.3%)	N/A
HLF	N/A	0 (0%)	0 (0%)	N/A
iAMP21	N/A	0 (0%)	0 (0%)	N/A
IKZF1 N159Y	N/A	0 (0%)	0 (0%)	N/A
KMT2A	0 (0%)	0 (0%)	0 (0%)	36 (0.6%)
KMT2A-like	N/A	0 (0%)	0 (0%)	N/A
Low hypodiploid	0 (0%)	0 (0%)	0 (0%)	47 (0.8%)
MEF2D	N/A	1 (3.1%)	1 (3.1%)	N/A
Near haploid	0 (0%)	0 (0%)	0 (0%)	N/A
NUTM1	N/A	0 (0%)	0 (0%)	N/A
PAX5 P80R	N/A	0 (0%)	0 (0%)	N/A
PAX5alt	N/A	2 (6.3%)	2 (6.3%)	N/A
Ph	0 (0%)	0 (0%)	0 (0%)	79 (1.3%)
Ph-like	N/A	3 (9.3%)	3 (9.4%)	N/A
TCF3::PBX1	0 (0%)	1 (3.1%)	1 (3.1%)	N/A
ZNF384	N/A	1 (3.1%)	1 (3.1%)	N/A
Not done/no results	10 (31.2%)	0 (0%)	0 (0%)	3,836 (62%)

^a^ One sample was classified as DUX4 (ALLSorts) and as Hyperdiploid (karyotyping). We categorized it as DUX4/Hyperdiploid. consequently, the total number of samples in DUX4 and Hyperdiploid subtypes is one less than expected, as this sample was counted in the DUX4/Hyperdiploid category. N/A: not available; na: not applicable.

Given the difficulty in evaluating the accuracy of subtype predictions in the samples that remained unclassified by traditional molecular and cytogenetic diagnosis, we used an additional subtype classifier, ALLCatchR (Beder et al., 2023). Overall, we found an 85.2% agreement with ALLsorts (23 out of 27 classified samples) (Figure 1B); and 100% agreement for the 19 “high confidence” predictions (Supplementary Material S1). ALLCatchR was unable to classify five samples (15.6%).

### Study of fusion transcripts

We used STAR-Fusion to detect and quantify fusion transcripts in RNA-seq data, and identified 82 chimeric transcripts: 19 (23%) inter-chromosomal, and 63 (77%) intra-chromosomal with an intergenic distance that ranged from <100 kb to >1 Mb (Supplementary Material S1), suggesting intra-chromosomal deletions. Eight of these fusions were previously reported in ALL (*EP300::ZNF384, ETV6::RUNX1, PAX5::ETV6, TCF3::FLI1, TCF3::PBX1, P2RY8::CRLF2, DUX4::IGH, MEF2D::BCL9*) and associated with prognostic molecular subtypes (Gu et al., 2019) (Figure 1A). Because STAR-Fusion requires read alignment to the whole genome, it demands substantial computational resources and processing time, often limiting its use in daily diagnosis settings. Therefore, we complementary applied RNAmut, a tool designed to detect cancer-specific mutations and fusions from RNA-seq data (Gu et al., 2020). This tool requires a pre-built index including a list of genes to be analyzed, requiring less computational resources and processing time. These features make it a potentially valuable tool for clinical applications. On the other hand, RNAmut is unable to analyze mutations and fusions in genes not included in the index, non-coding transcripts, transcripts without consensus CDS, and highly variable regions such as *IGH* genes. Therefore, only 24 of the 82 fusions detected by STAR-Fusion were evaluable by RNAmut. We built a RNAmut index including these 24 RNAmut-evaluable fusions and 77 additional fusions reported in the literature (Supplementary Material S1). Overall, the agreement between RNAmut and STAR-Fusion was low (8/37 in 32 patients, 21.6%). However, the concordance increased when we considered only the fusions previously reported in ALL (28/32, 87%, Figure 1C; Supplementary Material S1). RNAmut did not detect *MEF2D::BCL9*, whereas STAR-Fusion failed to detect *ETV6::RUNX1* in 2 out of 3 samples (validated by RT-PCR) (Figure 1C), suggesting that combination of tools can improve sensitivity. Prioritization of fusion transcripts was based on those detected by both tools, combined with fusion transcripts previously reported in ALL that were detected by only one tool but validated by specific gene expression profiles or RT-PCR, as described in the following sections.

### Identification of chimeras with long non-coding RNA partners, pseudogenes and novel fusion transcripts at the RNA level

After visually reviewing the mapped reads of fusion transcripts identified by STAR-Fusion, we identified 13 chimeras involving long non-coding RNAs. Among these, only the *ERG::LINC01423* fusion was highly expressed (65 reads spanning the junction), possibly originated from a 112,000 bp deletion on chromosome 21 that would lead to a partial deletion of *ERG* encompassing exons 2 to 10 (NM_182918.4) (Supplementary Material S2). A similar fusion involving these two genes has been previously reported in the DUX4 subtype in B-ALL patients (Migita et al., 2023). In agreement with this, ALLsorts predicted a DUX4 subtype for the patient with the *ERG::LINC01423* fusion.

**FIGURE 2:**
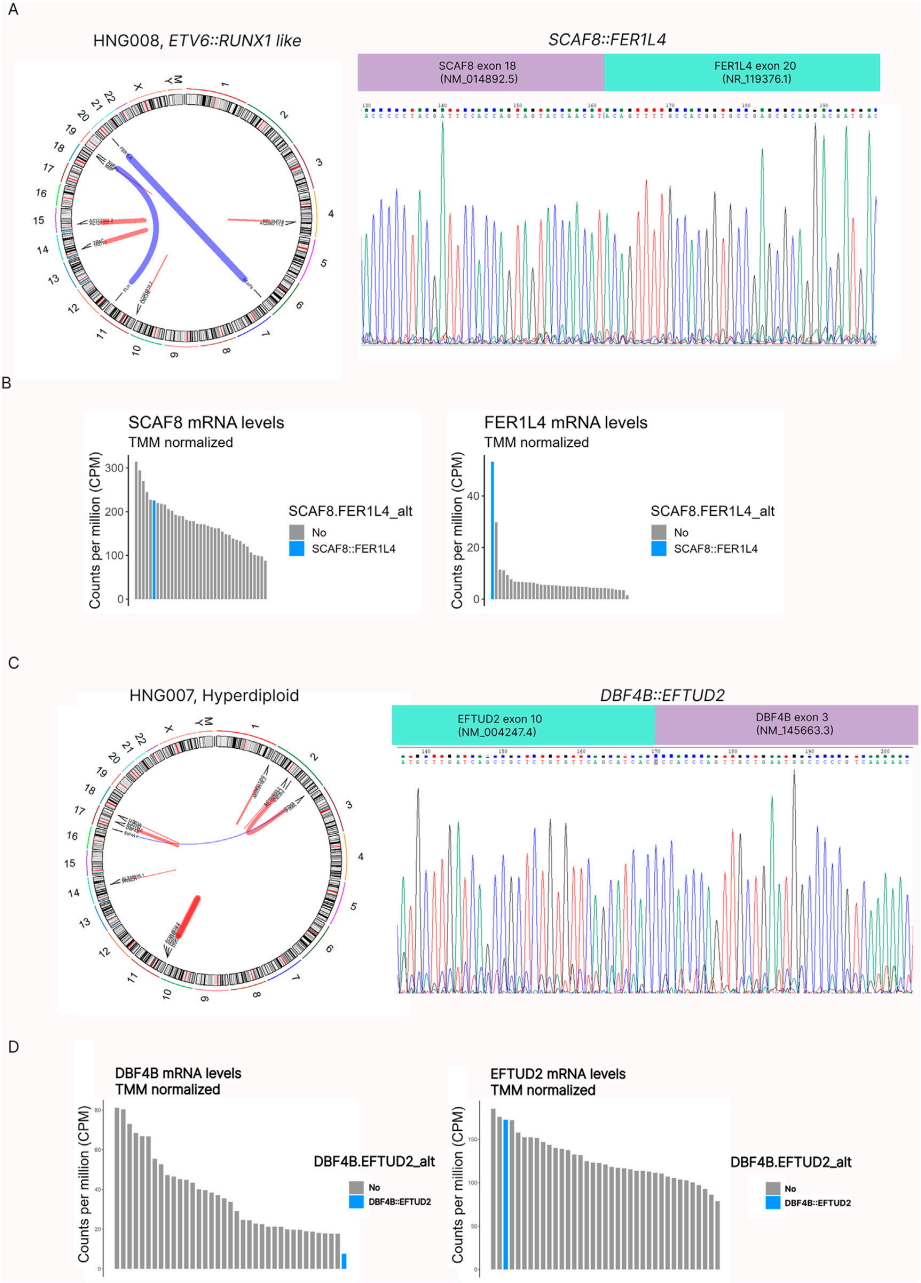
Identification and confirmation of novel fusion transcripts at the RNA level. **(A)** Circos Plot from a patient classified as ETV6::RUNX1-like. This patient had two translocations (blue lines): t (6; 20) *SCAF8::FER1L4* and t (11; 19) *TCF3::FLI*; and four intrachromosomal rearrangements (red lines). *SCAF8::FER1L4* novel fusion transcript involved exon 18 of *SCAF8* and exon 20 of *FER1L4*, as confirmed by Sanger sequencing. **(B)** Analysis of *FER1L4* and *SCAF8* mRNA levels by RNA-seq in the sample harbouring *SCAF8:FER1L4* compared to other patients. This study suggests an overexpression of *FER1L4* levels (Z-score = 5.1). **(C)** Circos Plot from a patient classified as hyperdiploid. This patient had the *DBF4B::EFTUD2* fusion (intrachromosomal, chromosome 17) and multiple additional intrachromosomal rearrangements. This novel fusion transcript involved exon 3 of *DBF4B* and exon 10 of *EFTUD2*, as confirmed by Sanger sequencing. **(D)** *DBF4B* mRNA levels by RNA-seq suggest mRNA levels by RNA-seq suggest *DBF4B* expression loss.

We also identified one inter-chromosomal fusion involving the *SCAF8* protein-coding gene and the pseudogene *FER1L4* (*SCAF8::FER1L4*; Figure 2A) with 17 reads spanning the breakage points. Because this was a novel fusion, we further analyzed the expression of the two partners. We observed that *FER1L4* was overexpressed (Z-score = 5.1), while *SCAF8* expression was not significantly altered (Z-score = 0.9) (Figure 2B). We detected *SCAF8::FER1L4* in co-occurrence with the *TCF3::FLI1* fusion -t (11; 19)- previously reported as oncogenic driver in pediatric B-lymphoblastic leukemia/lymphoma (Rowsey et al., 2019). These fusions were found in a 14-year-old patient diagnosed with an intermediate-risk B-ALL according to the ALLIC-GATLA-2010 guidelines, 63% of blasts in the bone marrow, and classified as ETV6::RUNX1-like by ALLSorts.

Finally, STAR-Fusion and RNAmut detected two novel fusions involving protein-coding genes. The *DBF4B::EFTUD2* fusion was detected with a medium/low sequencing depth (8 reads spanning the junction) and would be the consequence of a ∼110,000 bp intra-chromosomal deletion in chromosome 17. This fusion joins exon 3 of *DBF4B* to exon 10 of *EFTUD2* (Figure 2C) and results in an in-frame chimera. It was identified in a 2-year-old patient with hyperdiploid subtype and classified as intermediate-risk according to the protocol guidelines. This patient also harbored a SNV in *CSF3R* (p.G147R, described in the following section). The mRNA levels of *DBF4B* suggest a nearly-null expression of wild-type *DBF4B* (Z-score = −1.5) while *EFTUD2* expression was among the highest in this cohort (Z-score = 1.8) (Figure 2D).

The other novel fusion, *ABHD17B::CEMIP2*, would be the consequence of a ∼150,000 bp intra-chromosomal deletion in chromosome 9. We detected this fusion in 5 patients, with low abundance (3–6 reads), and it was detected by both tools in only one patient. Given the low concordance and expression level, we did not further analyze this fusion.

Finally, we detected chimeric genes in all 32 samples with variable sequencing depth (Supplementary Material S2, S3). Most fusions were intra-chromosomal, likely resulting from chromosomal deletions.

### Identification of SNVs and small InDels by transcriptome analysis

We ran RNAmut with a custom index of 114 genes commonly mutated in pediatric ALL to identify SNVs and small InDels (Supplementary Material S1). This analysis detected 9,594 variants across the 32 patients. After annotation and filtering, we identified 21 different SNVs/InDels in 16 genes across 14 patients (42%) (Figure 2). Based on the OncoKB classification, these SNVs/InDels included 5 oncogenic variants (NSD2 p. E1099K, PTPN11 p. D61F, FLT3 p. N676K, FLT3 p. D835E, and NRAS p. G12D), 7 likely oncogenic variants (ATM p. P604S in two patients, NRAS p. G12S, KRAS p. Q61P, TP53 p. P152L, TP53 p. R267P, NF1 p. R1306*, TCF3 p. S338fs*10), and 9 unclassified variants (PAX5 p. P34L, PAX5 p. R38C, ZEB2 p. H1038R, IKZF1 p. D186Y, CREBBP p. G1542V, DUX4 p. I65N, CSF3R p. G147R, ETV6 p. Q12del, FLT3 R833-D834_Ins:S). Of these, 20 variants were confirmed using CTAT-Mutations, a bioinformatic tool designed for detecting variants from whole-transcriptome data (https://github.com/NCIP/ctat-mutations) (Supplementary Material S1); 3 of them were novel, while the remaining 17 had been previously reported in variant databases (Supplementary Material S1). The variant detected only by RNAmut (ETV6 p. Q12del) is compatible with an alternative splicing isoform of *ETV6*.

**FIGURE 3:**
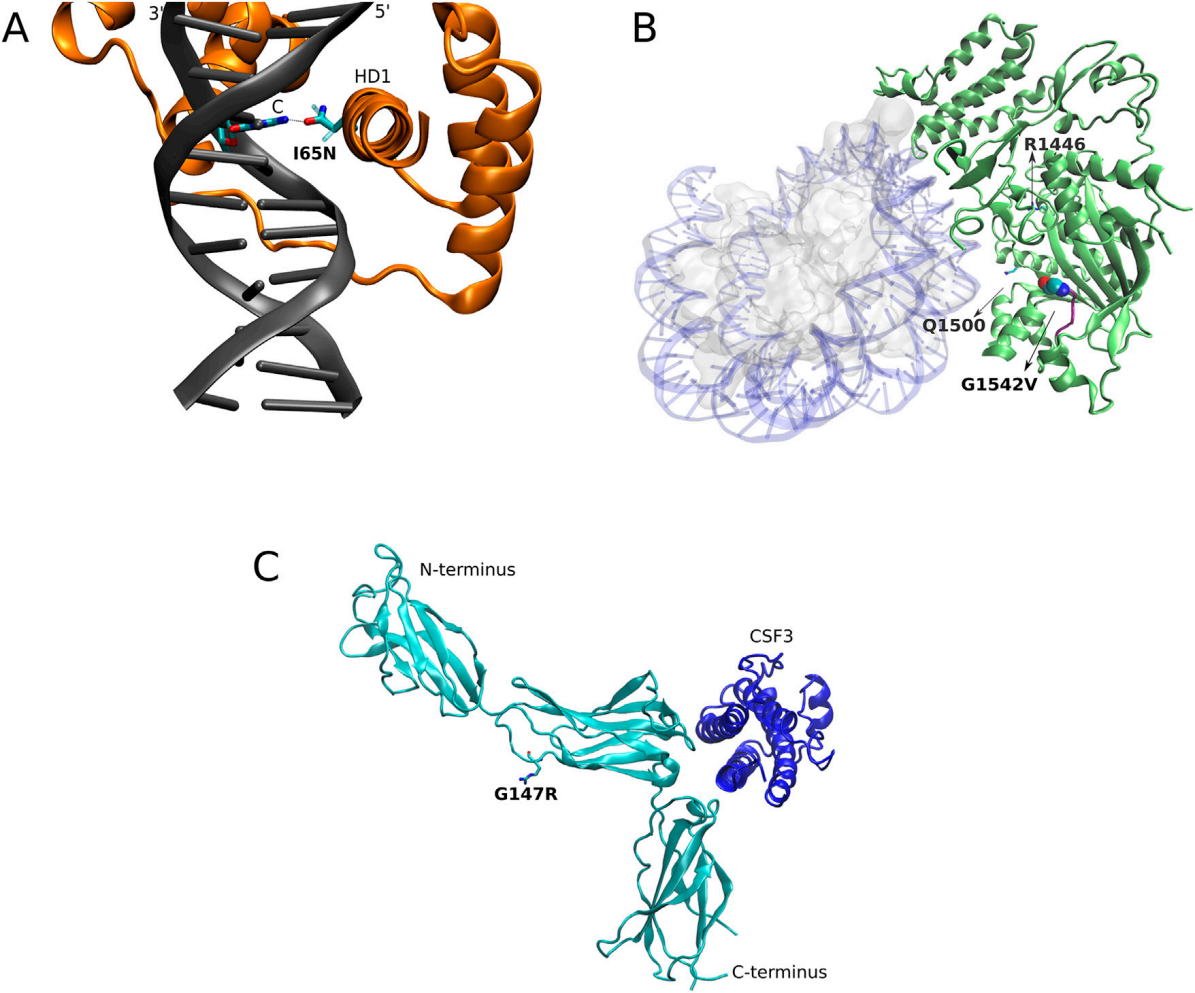
Structural analysis of novel single nucleotide variants. Superimposition of wildtype structure with mutated residues in the described variants are shown. **(A)** DUX4 a3 helix of HD1 domain inserted in the DNA major groove, with the side chain of residue 65 making base-specific contacts (sticks). Hydrogen bonds are depicted by a dotted line. Note that, while Ile65 in the wild-type (ghost licorice side-chain) DUX4 version does not directly interact with any base, Asn65 variant (solid licorice side-chain) offers new interactions with the base, affecting the original interactions, since the N in the base is now able to form hydrogen bond interactions with the Asn side chain groups, as depicted. PDBid: 5Z6Z. **(B)** Cryo-EM structure (PDBid: 8HAL) of the CBP catalytic core of CREB-binding protein (green) bound to histone H2B and acetylated H4 (ghost gray) and DNA (transparent blue). As noted, G1542V variant occurs in a disordered region (purple) connecting two ɑ-helices. While not directly located in the protein-histone complex interface, the variant might compromise the stability of this region, as also reported for structurally near variants identified by Mullighan et al. (2011). **(C)** CSF3R (cyan) interacting with CSF3 ligand (blue). As noted, while G147R variant (R side chain overlaps with the short G side chain, which is not visible) does not directly affect the intermolecular interaction with CSF3, but it might be responsible for: a destabilization of the loop, an alteration in the interaction with other molecules, or a combination of both. PDBid: 2D9Q.

Next, we assessed the potential impact of the 3 novel variants (DUX4 p. I65N, CREBBP p. G1542V, and CSF3R p. G147R) using *in silico* methods. All these variants were located in highly conserved residues, suggesting a possible alteration of protein function (Table 3; Figure 3; Supplementary Material S2, S4). DUX4 is a transcription factor involved in the regulation of critical genes for early development. The variant p. I65N is located in homeodomain 1, and the residue change likely alters the interaction between DUX4 and its target DNA (Figure 3A), potentially affecting its transcriptional activity.

**TABLE 3:** Classification of novel single nucleotide variants.

Gene	Localization	Variant	Reads var/wt (VAF^a^)	Type of variant	Franklin	Cosmic	Cancer genome interpreter
*CREBBP*	HAT (histone acetylase protein) domain	NM_004380.3:c.5422G>T; NP_004371.2: p.Gly1542Val	39/0 (1)	missense	TIER3, VUS	NA, G1542S, G1542D	Driver
*CSF3R*	extracellular domain, loop	NM_000760.4:c.439G>C; NP_000751.1: p.Gly147Arg	92/82 (0.52)	missense	TIER2, VUS	1 report in basal cell carcinoma	Driver
*DUX4*	homeodomain, DNA interaction site	NM_001306068.3:c.194T>A; NP_001292997.1: p.Ile65Asn	657/0 (1)	missense	TIER3, VUS	No substitutions in DUX4	NA

^a^ in RNA-seq. VAF: variant allele frequency; VUS: variant of uncertain significance; NA: not available.

CREBBP regulates cell growth, division, maturation and differentiation. The novel variant p. G1542V is located in the histone acetyltransferase domain, a known mutation hotspot in relapsed ALL (Mullighan et al., 2011). The p. G1542 residue lies in a disordered region, and the substitution to valine might compromise domain stability and/or interactions with other molecules (Figure 3B).

CSF3R participates in granulopoiesis during inflammation and in cell surface adhesion or recognition processes. The p. G147R variant is located in the receptor’s extracellular region. Although not predicted to directly interfere with the interaction with the ligand CSF3, the substitution of a small uncharged glycine with a larger positive-charged arginine might destabilize the loop, modify the interaction with other molecules, or both (Figure 3C).

### Molecular profiling confirms the accuracy of the bioinformatic subtyping

Whole-transcriptome sequencing provides an integrated view of fusion transcripts, SNVs/InDels and gene expression, allowing for deep molecular subtyping. In order to confirm the accuracy of the bioinformatic subtyping tools used, we analyzed the key biomolecules defining the different ALL subtypes.

**FIGURE 4:**
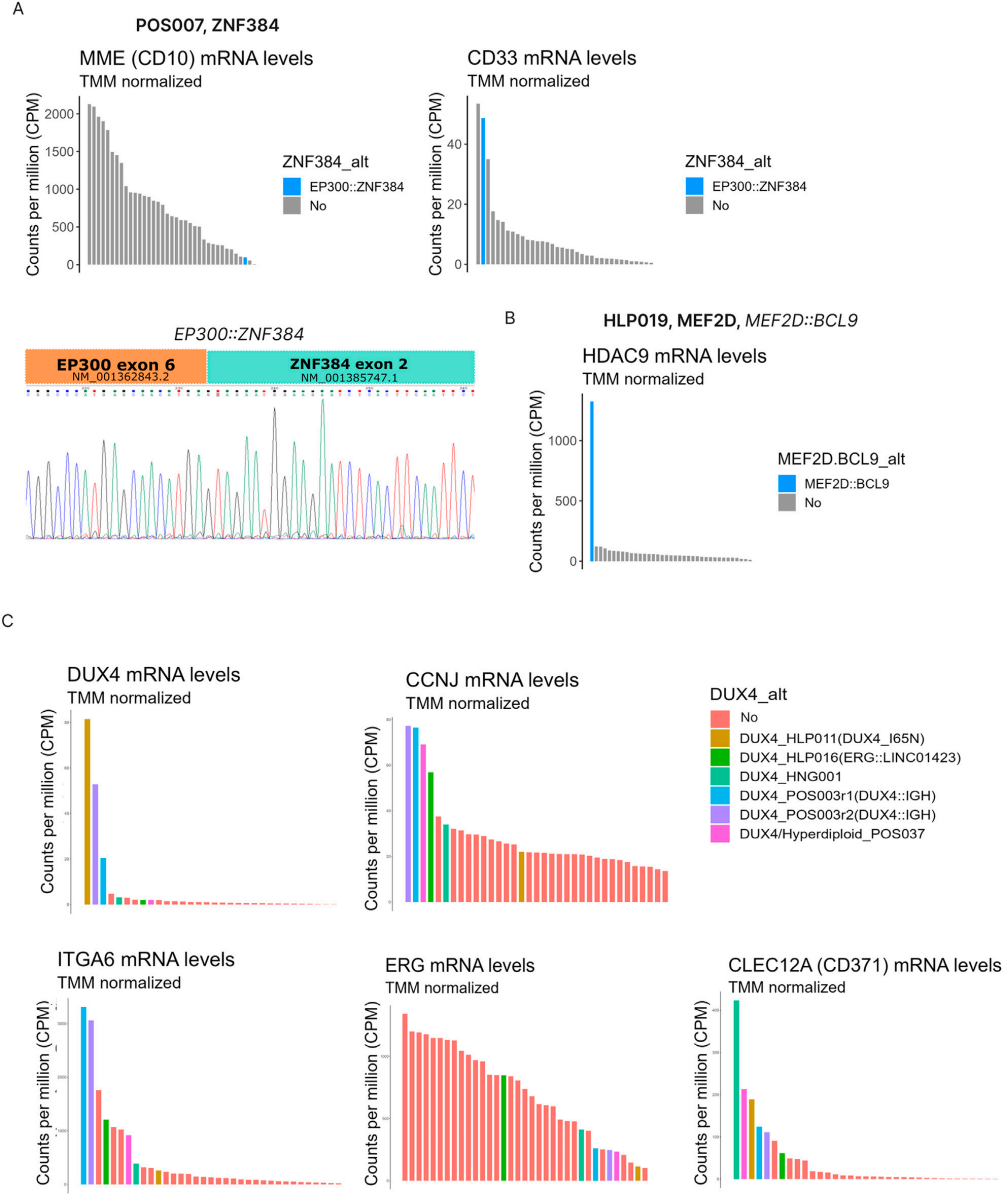
Integration of multiple molecular data confirms the accuracy of the bioinformatic subtyping. ZNF384 subtype was predicted in a patient harboring *EP300::ZNF384* fusion transcript, reduced abundance of *CD10* and increased *CD33* **(A)**. MEF2D was predicted in a patient harboring *MEF2D::BCL9* fusion transcript and increased levels of *HDAC9* mRNA **(B)**. DUX4 subtype was predicted in 5 patients with different molecular features. In some but not all patients there was an association with upregulation of *DUX4::IGH* target genes (*ITGA6*, *CCNJ*), increased levels of *DUX4* and *CD371*, and reduced levels of *ERG* **(C)**.

The patient classified as ZNF384-alt by ALLSorts harbored the *EP300::ZNF384* fusion transcript and a gene expression profile consistent with the expected immunophenotype: low CD10 (Z-score = −1.14) and high CD33 expression (Z-score = 3.3) (Figure 4A).

The patient classified as MEF2D had, as expected, a fusion involving MEF2D (*MEF2D9::BCL9*) and high expression of *HDAC9* (Figure 4B). Although classified as intermediate-risk following the ALLIC-BFM criteria, the patient relapsed at 11 months and died at 16 months, reflecting the poor prognosis associated with this subtype despite an initial good clinical response (MRD < 0.1% at days 8 and 15).

ALLSorts identified five patients as DUX4; however, the canonical *DUX4::IGH* fusion transcript was detected only in one sample. As expected, this patient had high expression of *DUX4*, *CCNJ* and *ITGA6*; being the last two direct transcriptional targets of *DUX4::IGH* (Figure 4C). In addition, this patient had mutations in *ZEB2*, *TP53*, and *PTPN11* (Figure 1). Another patient had the novel DUX4 p. I65N variant (Table 3), located within the homeodomain coding region as described in the previous section (Figure 3). This patient showed overexpression of *DUX4*; however, we observed, as predicted by the *in silico* analysis, normal levels of *CCNJ* and *ITGA6* (Figure 4C) suggesting a distinct expression profile compared to the *DUX4::IGH* samples. Another patient carried an intra-chromosomal *ERG* fusion transcript, indicative of a 112,000 bp deletion in chromosome 21 leading to partial *ERG* loss, a common alteration in the DUX4 subtype. Although no canonical *DUX4* alterations were detected in the remaining cases, all five DUX4 samples had high expression of *CD371* (above quartile 75%), a marker associated with this subtype and linked to lineage switching (Figure 4C).

### Association of molecular alterations with clinical variables

We did not detect significant associations between molecular subtypes and the clinical variables relapse, MRD at day 15, death, response to prednisone at day 8, severe acute toxicity related to treatment, sex nor age. However, given the small sample size in each subtype and follow-up time (Table 1), we cannot rule out the existence of associations that cannot be detected in this small cohort.

Four of the five patients who relapsed were classified as B-other ALL and one as hyperdiploid following the ALLIC-GALTA-2010 guidelines. Among them, one was diagnosed with high-risk ALL and MRD > 10%, one with intermediate-risk disease and MRD > 10%, and three with intermediate-risk and MRD < 10%. The patient with intermediate-risk disease and MRD > 10% harbored a truncating mutation in *NF1* and the novel mutation CREBBP p. G1542V. Notably, all three intermediate-risk patients with MRD < 10% had high-risk molecular alterations or subtypes (Tables 4, 5): NSD2 p. E1099K, *MEF2D::BCL9*/ATM p. P604S, or PAX5alt subtype NRAS p. G12S. These findings suggest the potential of comprehensive molecular subtyping to refine risk stratification and guide precision medicine.

**TABLE 4:** Analysis of relapse stratified by molecular subtypes and genetic alterations.

		Relapse		p-value
		No; n = 27	Yes; n = 5	
**Follow-up time** median (IQR)		31 (25–35)	34 (16–50)	0.70^a^
**Molecular subtype^b^**				0.24^c^
	**Genetic alteration(s)**			
Hyperdiploid	ABHD17B::CEMIP2^d^	1 (3.7%)	0 (0.0%)	
	CSF3R p.G147R + DBF4B::EFTUD2^d^	1 (3.7%)	0 (0.0%)	
	FLT3 p.N676K	1 (3.7%)	0 (0.0%)	
	FLT3 p.D835E + R833-D834_Ins:S	1 (3.7%)	0 (0.0%)	
	Hyperdiploid	6 (22.2%)	0 (0.0%)	
	NRAS p.G12D + PAX5 p.R38C	1 (3.7%)	0 (0.0%)	
	NF1 p.R1306* + CREBBP p.G1542V	0 (0.0%)	1 (20.0%)	
	DUX4^e^ + TP53 p.R267P + IKZF1 p.D186Y	0 (0.0%)	1 (20.0%)	
	total	11 (40.7%)	2 (40.0%)	
ETV6::RUNX1	ETV6::RUNX1	2 (7.4%)	0 (0.0%)	
	ETV6::RUNX1 + ATM p.P604S	1 (3.7%)	0 (0.0%)	
	total	3 (11.1%)	0 (0.0%)	
ETV6::RUNX1-like	**NSD2 p.E1099K**	0 (0.0%)	1 (20.0%)	
	TCF3:FLI1 + SCAF8::FER1L4^d^	1 (3.7%)	0 (0.0%)	
	total	1 (3.7%)	1 (20.0%)	
Ph-like	KRAS p.Q61P	1 (3.7%)	0 (0.0%)	
	Ph-like	1 (3.7%)	0 (0.0%)	
	P2RY8::CRLF2	1 (3.7%)	0 (0.0%)	
	total	3 (11.1%)	0 (0.0%)	
PAX5alt	NRAS p.G12S + PAX5 p.P34L	0 (0.0%)	1 (20.0%)	
	PAX5::ETV6	1 (3.7%)	0 (0.0%)	
	total	1 (3.7%)	1 (20.0%)	
DUX4	DUX4	1 (3.7%)	0 (0.0%)	
	DUX4::IGH + PTPN11 p.D61Y + TP53 p.P152L + ZEB2 p.H1038R	1 (3.7%)	0 (0.0%)	
	DUX4 p.I65N + ETV6_Q12del	1 (3.7%)	0 (0.0%)	
	ERG::LINC01423	1 (3.7%)	0 (0.0%)	
	total	4 (14.8%)	0 (0.0%)	
MEF2D	**MEF2D::BCL9** + ATM p.P604S	0 (0.0%)	1 (20.0%)	
TCF3::PBX1	TCF3::PBX1 + TCF3_S338fs*10	1 (3.7%)	0 (0.0%)	
ZNF384	EP300::ZNF384	1 (3.7%)	0 (0.0%)	
B-other	Unclassified	2 (7.4%)	0 (0.0%)	

^a^ Wilcoxon rank sum test.

^b^ Final classification.

^c^ Fisher’s exact test (by molecular subtype).

^e^ Classified as DUX4/Hyperdiploid.

^d^ Novel fusion transcripts.

In bold are depicted the high-risk alterations identified in patients initially classified as intermediate-risk according to the protocol guidelines.

**TABLE 5:** Analysis of minimal residual disease at day 15 by flow cytometry stratified by molecular subtypes and genetic alterations.

		MRD d15^a^			p-value
		<0.1% N = 11	0.1%–10% N = 14	>10% N = 5	
**Follow-up time** median (IQR)		28 (25–33)	35 (32–36)	31 (15–48)	0.20^b^
**Molecular subtype^c^**					0.90^d^
	**Genetic alteration(s)**				
Hyperdiploid	ABHD17B::CEMIP2^e^	0 (0.0%)	1 (7.1%)	0 (0.0%)	
	CSF3R p.G147R + DBF4B::EFTUD2^e^	1 (9.1%)	0 (0.0%)	0 (0.0%)	
	FLT3 p.N676K	1 (9.1%)	0 (0.0%)	0 (0.0%)	
	FLT3 p.D835E + R833-D834_Ins:S	1 (9.1%)	0 (0.0%)	0 (0.0%)	
	Hyperdiploid	0 (0.0%)	5 (35.7%)	0 (0.0%)	
	NRAS p.G12D + PAX5 p.R38C	1 (9.1%)	0 (0.0%)	0 (0.0%)	
	NF1 p.R1306* + CREBBP p.G1542V	0 (0.0%)	0 (0.0%)	1 (20.0%)	
	DUX4^f^ + TP53 p.R267P + IKZF1 p.D186Y	0 (0.0%)	0 (0.0%)	1 (20.0%)	
	total	4 (36.4%)	6 (42.9%)	2 (40.0%)	
ETV6::RUNX1	ETV6::RUNX1	1 (9.1%)	1 (7.1%)	0 (0.0%)	
	ETV6::RUNX1 + ATM p.P604S	0 (0.0%)	1 (7.1%)	0 (0.0%)	
	total	1 (9.1%)	2 (14.3%)	0 (0.0%)	
ETV6::RUNX1-like	**NSD2 p.E1099K**	0 (0.0%)	1 (7.1%)	0 (0.0%)	
	TCF3::FLI1 + SCAF8::FER1L4^e^	1 (9.1%)	0 (0.0%)	0 (0.0%)	
	total	1 (9.1%)	1 (7.1%)	0 (0.0%)	
Ph-like	KRAS p.Q61P	1 (9.1%)	0 (0.0%)	0 (0.0%)	
	Ph-like	0 (0.0%)	0 (0.0%)	1 (20.0%)	
	P2RY8::CRLF2	NA			
	total	1 (9.1%)	0 (0.0%)	1 (20.0%)	
PAX5alt	NRAS p.G12S + PAX5 p.P34L	1 (9.1%)	0 (0.0%)	0 (0.0%)	
	PAX5::ETV6	0 (0.0%)	1 (7.1%)	0 (0.0%)	
	total	1 (9.1%)	1 (7.1%)	0 (0.0%)	
DUX4	DUX4	0 (0.0%)	1 (7.1%)	0 (0.0%)	
	DUX4::IGH + PTPN11 p.D61Y + TP53 p.P152L + ZEB2 p.H1038R	0 (0.0%)	0 (0.0%)	1 (20.0%)	
	DUX4 p.I65N + ETV6_Q12del	1 (9.1%)	0 (0.0%)	0 (0.0%)	
	ERG::LINC01423	0 (0.0%)	1 (7.1%)	0 (0.0%)	
	total	1 (9.1%)	2 (14%)	1 (20.0%)	
MEF2D	**MEF2D::BCL9** + ATM p.P604S	1 (9.1%)	0 (0.0%)	0 (0.0%)	
TCF3::PBX1	TCF3::PBX1 + TCF3_S338fs*10	0 (0.0%)	1 (7.1%)	0 (0.0%)	
ZNF384	EP300::ZNF384	0 (0.0%)	1 (7.1%)	0 (0.0%)	
B-other	Unclassified	1 (9.1%)	0 (0%)	1 (20.0%)	

^a^ two patients had missing information on MRD (1 hyperdiploid and 1 Ph-like).

^b^ Kruskal–Wallis rank sum test.

^c^ Final classification.

^d^ Fisher’s exact test (by molecular subtype).

^f^ Classified as DUX4/Hyperdiploid.

^e^ Novel fusion transcripts.

In bold are depicted the high-risk alterations identified in patients initially classified as intermediate-risk according to the protocol guidelines.

## Discussion

Acute lymphoblastic leukemia is the most common pediatric cancer, and its treatment success increasingly depends on precise molecular characterization. In this study, we conducted a comprehensive molecular characterization of childhood B-ALL in Argentine patients using whole-transcriptome sequencing on bone marrow aspirates at diagnosis. Identifying ALL genetic driver lesions and molecular subtypes at diagnosis is crucial for accurate risk stratification and treatment decision-making. Combining the identification of genetic subtypes with measurement of minimal residual disease during treatment allows a refinement in treatment intensity, which can contribute to reducing the rates of induction death, achieving better disease control in high risk patients, or benefiting from the addition of molecularly targeted therapy, immunotherapy, or both (Jeha et al., 2021). However, its implementation, particularly in Latin American countries, remains challenging. The increasing number of ALL molecular subtypes and the diversity of genetic alterations demand large resources, including specialized personnel, infrastructure, funding and time, which are often limited in this region. For instance, the ALLIC-BFM-2009 protocol, which recruited 6,187 patients from 13 resource-limited countries, reported that over 60% of patients lacked cytogenetic and/or molecular results, leading to incomplete molecular characterization of the disease (Campbell et al., 2023). Furthermore, most molecular subtyping tools were primarily developed using cohorts from the U.S. and Western Europe, raising concerns about their classification accuracy and clinical relevance in populations with different genetic background and environmental exposures. Previous studies have also shown that therapeutic response rates are highly variable and tend to be lower in patients from low- and middle-income countries (Chatenoud et al., 2010; Allemani et al., 2018; Lee et al., 2022).

Expanding molecular and genetic subtyping in underrepresented populations will require improved access to technical and financial resources. In this context, our study is the first to provide a transcriptome-based molecular profiling in Argentine patients. The sample size of this study limits the comparison of molecular subtype frequencies with cohorts from other regions. However, when we compared the most frequent subtypes with the patients treated at St. Jude Children’s Research Hospital (Brady et al., 2022), we observed a higher frequency of DUX4 (13% vs. 3.6%) and hyperdiploid (38% vs. 25%) subtypes, and a lower frequency of ETV6::RUNX1 (9.4% vs. 19%). A previous work by Coccé et al. subtyped 847 B-ALL patients from Argentina using cytogenetics, FISH and RT-PCR, and reported 15.2% ETV6::RUNX1 and 34.7% hyperdiploidy (Coccé et al., 2015). Lower prevalence of ETV6::RUNX1 subtype in populations other than caucasian has been reported by independent studies (Aldrich et al., 2006; Bekker-Méndez et al., 2014; Yin et al., 2022). The use of whole transcriptome sequencing on diagnostic bone marrow aspirates enabled the integrative assessment of multiple molecular alterations, including gene expression profiles, small sequence variations, and gene fusions. These findings add significant clinical value, offering potential improvements in risk stratification and the identification of therapeutic targets, particularly in cases where traditional methods fail to define a molecular subtype.

The study of fusion genes is critical for accurate ALL diagnosis, prognosis, and risk stratification. In this study we used STAR-Fusion, a tool that demands high computational resources, and RNAmut, which requires a pre-defined list of protein-coding genes, resulting in the need of fewer computational resources. We observed that both tools failed to detect all fusions, and the overall agreement between them was low. Our results suggest a lower sensitivity for RNAmut given that the detected fusion transcripts had an average 4.5-fold higher expression levels than those missed by the tool (Supplementary Material S1). Similarly, Vicente Garces et al. found that none of the five fusion-calling pipelines tested achieved perfect sensitivity and precision (Vicente-Garcés et al., 2023). These findings underscore that there is room for improvement in fusion transcript detection tools from RNA-seq and/or the use of multiple fusion-detection tools to improve the detection of chimeric genes.

We also confirmed that the analysis of gene expression patterns from RNA-seq data can provide additional supporting evidence to validate the molecular subtype and the presence of fusion genes. For example, in our study, while STAR-Fusion detected the *MEF2D::BCL9* chimera, RNAmut did not. To resolve this discrepancy, we analyzed the expression of *HDAC9*, as it has been reported that cells and patients with the *MEF2D::BCL9* fusion have a markedly high expression of HDAC9 (Suzuki et al., 2016; Ohki et al., 2019). As expected, we observed high *HDAC9* expression in this patient, supporting the classification as MEF2D subtype and the presence of *MEF2D::BCL9*.

RNAmut is a user-friendly tool originally developed for acute myeloid leukemia. To our knowledge, our study is the first application of RNAmut to ALL samples. We constructed a custom gene index aimed at identifying clinically relevant SNVs, InDels and gene fusions in ALL. This allowed us to identify novel SNVs in genes previously reported as mutated in ALL or other hematological malignancies: *CREBBP*, *CSF3R* and *DUX4*. *In silico* modeling of the novel SNVs suggested a possible disruption of normal protein folding and/or activity given the localization of variant residues and the changes in their biochemical properties. The SNV in CREBBP (p.G1542V) was found in one patient classified as intermediate-risk, who relapsed at 33 months; and was found in co-occurrence with a truncating oncogenic mutation in *NF1*. In agreement with this, *CREBBP* mutations have been previously associated with a high incidence of relapse in ALL patients (Mullighan et al., 2011), whereas *NF1* mutations do not seem to change overall survival in ALL (Huang et al., 2024).

The *CSF3R* p. G147R variant was identified in an intermediate-risk patient with a hyperdiploid subtype and MRD < 0.1% at day 15, co-occurring with the novel *DBF4B::EFTUD2* fusion. *In silico* analysis suggests that the variant found in *CSF3R* could potentially affect protein stability or binding of extracellular molecules. While *CSF3R* mutations are rare in B-ALL, truncating and activating mutations in this gene are known drivers in chronic neutrophilic leukemia and atypical chronic myeloid leukemia. In addition, mutations in the intracellular domain of CSF3R have been associated with specific therapeutic vulnerabilities, including sensitivity to dasatinib and resistance to ruxolitinib, suggesting potential clinical relevance (Maxson et al., 2013; Schwartz and Wieduwilt, 2020; Trottier et al., 2020).

The novel chimera *DBF4B::EFTUD2* results from an in-frame fusion of two protein-coding genes. DBF4B is a key regulator of CDC7 kinase, which is involved in DNA replication and response to replication stress. Although the biological function of the chimeric protein remains unknown, the patient exhibited near-null expression of wild-type DBF4B, potentially disrupting replication processes, as previously reported in DBF4-deficient cells (Göder et al., 2024). The fusion partner EFTUD2 is a spliceosomal GTPase. Notably, we detected high expression of wild-type *EFTUD2* in this patient, consistent with its reported oncogenic role in hepatocellular carcinoma (Tu et al., 2020), and suggesting that *EFTUD2* may also contribute to leukemogenesis in ALL.

The third novel SNV we identified was DUX4 p. I65N, located within one of the protein’s homeodomains. Based on our *in silico* predictions, this variant may impair DUX4 transcriptional activity. Notably, the patient carrying this variant showed the highest expression of *DUX4* within our cohort (Hendrickson et al., 2017). Interestingly, previous studies have shown that overexpression of full-length or C-terminal-truncated DUX4 can be toxic to cells (Tian et al., 2019). This apparent discrepancy may be explained by the predicted impaired DNA-binding affinity of the DUX4 p. I65N variant, which could allow cells to tolerate high levels of full-length *DUX4* without experiencing toxic effects. The patient having this mutation was classified as intermediate-risk with MRD < 0.1%, had a DUX4 subtype, and has neither relapsed nor died as of the latest analyses, suggesting a milder disease. These *in silico* results warrant functional validation comparing wild type and variant proteins.

Finally, we identified a novel fusion involving a pseudogene/long non-coding RNA: *SCAF8::FER1L4*. This fusion is transcribed into a chimeric RNA of 6,907 nucleotides, comprising the N-terminal region of SCAF8 (714 amino acids), 11 amino acids from FER1L4, and a premature stop codon. *FER1L4* is annotated as a pseudogene in Ensembl (ENSG00000088340.11; last access: April 5th, 2025) but has been described as a long non-coding RNA by several studies. Initially identified in gastric cancer, *FER1L4* has since been reported to be an oncogene by modulating the PI3K/AKT pathway in various cancer cells, including osteosarcoma, hepatocellular carcinoma, and colon cancer. It plays critical roles in numerous processes such as cell growth, apoptosis, migration, and invasion (Mou et al., 2022). The biological activity of this novel fusion in ALL and other malignancies remains to be studied.

Altogether, RNA-seq analysis of leukemic bone marrow enabled molecular subtyping in 93.7% of B-ALL patients and facilitated the identification of both known and novel molecular alterations. Despite further validation in independent cohorts is required, this study adds valuable evidence and methodology to support transcriptome-based approaches in ALL molecular diagnostics.

## Data Availability

The datasets presented in this study can be found in online repositories. The names of the repository/repositories and accession number(s) can be found below: https://www.ebi.ac.uk/ena, PRJEB80172.
