# Supplementary material for: Genetic characterization of pediatric B-cell acute lymphoblastic leukemia in Argentina uncovers molecular heterogeneity and novel variants
Source: Front Pharmacol. 2025 Nov 17;16:1701680. doi: 10.3389/fphar.2025.1701680 (PMC12665890; doi:10.3389/fphar.2025.1701680)

**Supplementary Material S2 for article “Genetic Characterization of Pediatric B-cell Acute Lymphoblastic Leukemia in Argentina Uncovers Molecular Heterogeneity and Novel Variants”**

*Supporting Methods and Results*

*1. Pipeline for filtering output files from RNAmut analysis.*

1. Results from RNAmut are exported as a text file per index per sample, that includes information about Gene, Mutation, ProtMut, MutReads, WTReads, VAF and Info for each variant. Each index allows a maximum of 50 genes, therefore results from all the indexes are combined for each sample.
2. Exclusion of variants with VAF <0.2 and MutReads <10 (this step filters out fusion transcripts because their VAF=NA due to the absence of WT reads).
3. Conversion of text files into VCF format.
4. VCF files were submitted to the Ensembl webtool VEP (<https://www.ensembl.org/Tools/VEP>). VEP output files were filtered according to clinical significance (exclusion of benign variants), OR phenotype (“leukemia”) OR Polyphen classification (“damaging”), and only variants classified as TSL=1 OR TSL=2 (TSL=Transcript Support Level) were included.
5. Further filtering that included only variants with MutReads ≥ 15 was applied, in order to exclude variants that were present in all samples, which were suggestive of technical artifacts (*SUZ12, FOXP1, KMT2A, RANBP2*).
6. Individual variants were manually prioritized by comparing results from OncoKB, Varsite, COSMIC, Cancer Genome Interpreter.

*2. Detection of SNV/Indel and fusion transcripts by RT-PCR and Sanger sequencing.*

SNVs/InDels were confirmed by retrotranscription of patient samples (RevertAid, Thermo Fisher Scientific, USA; random primers) followed by PCR (DNA Taq polymerase Pegasus, PB-L, Argentina) and Sanger sequencing (Macrogen, Korea). Primer sequences are detailed in Supplementary Material S1. There was a high concordance between the Variant Allele Fraction (VAF) estimated by RNAmut and the relative proportion of peaks in Sanger sequencing (Fig. S1).

*Supplementary Figure S1. Confirmation of RNAmut-called variants (purple box) by RT-PCR followed by Sanger sequencing using cDNA from patient bone marrow aspirates.*


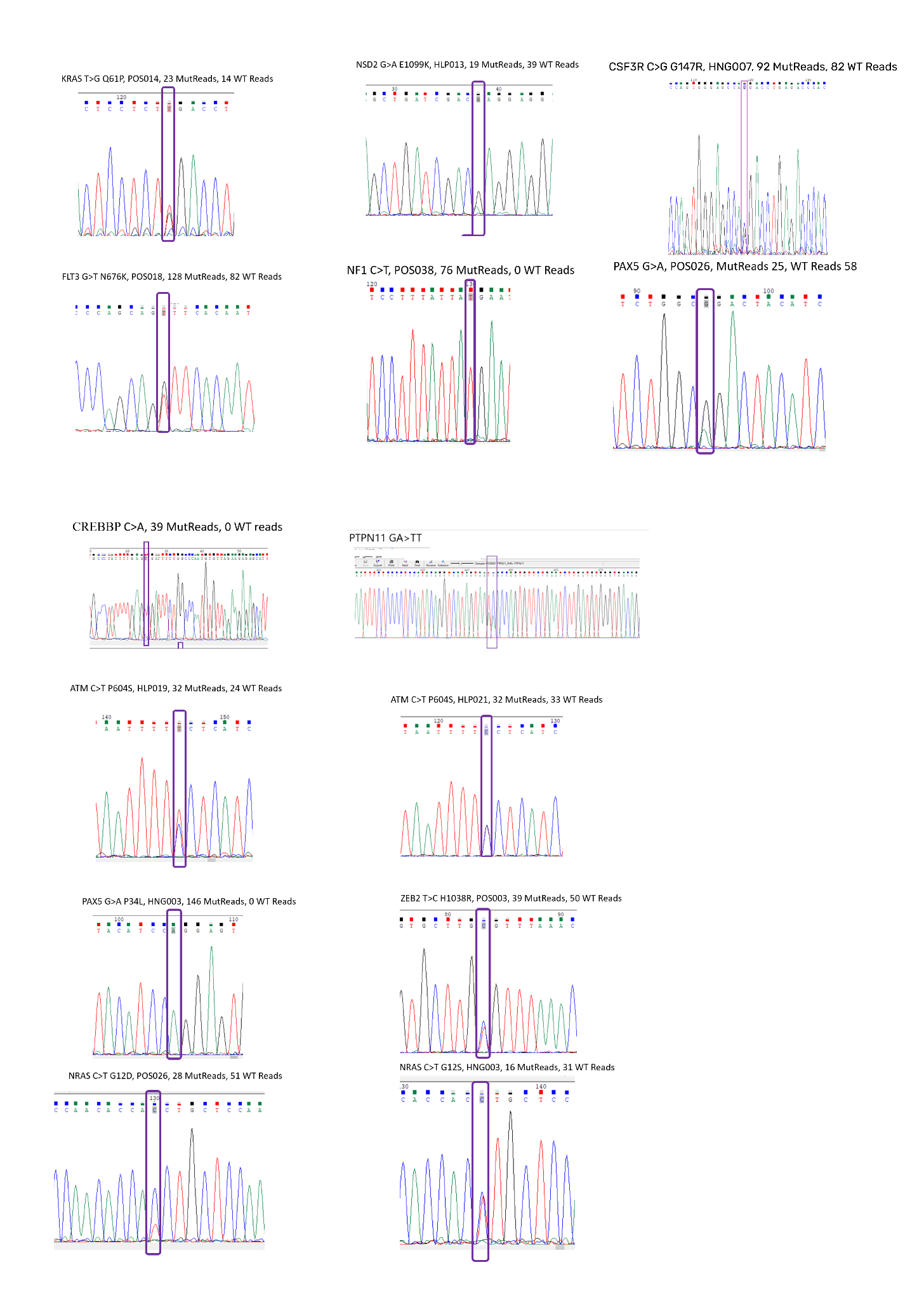


*3. Identification of chimeras including long non-coding RNA partners, pseudogenes and novel fusion transcripts.*

After visually reviewing the mapped reads of fusion transcripts identified by STAR-Fusion, we confirmed 13 chimeras involving long non-coding RNAs. Among these, only the *ERG::LINC01423* fusion was highly expressed (65 reads spanning the junction) (Fig. S2).

*Supplementary Figure S2. Circos Plot from patient sample with DUX4 subtype harboring a ERG::LINC01423 fusion (chr21) and additional intrachromosomal rearrangements. This fusion transcript would arise from a 112,000 bp deletion on chromosome 21 leading to a partial deletion of ERG (exons 2 to 10, NM_182918.4).*

**
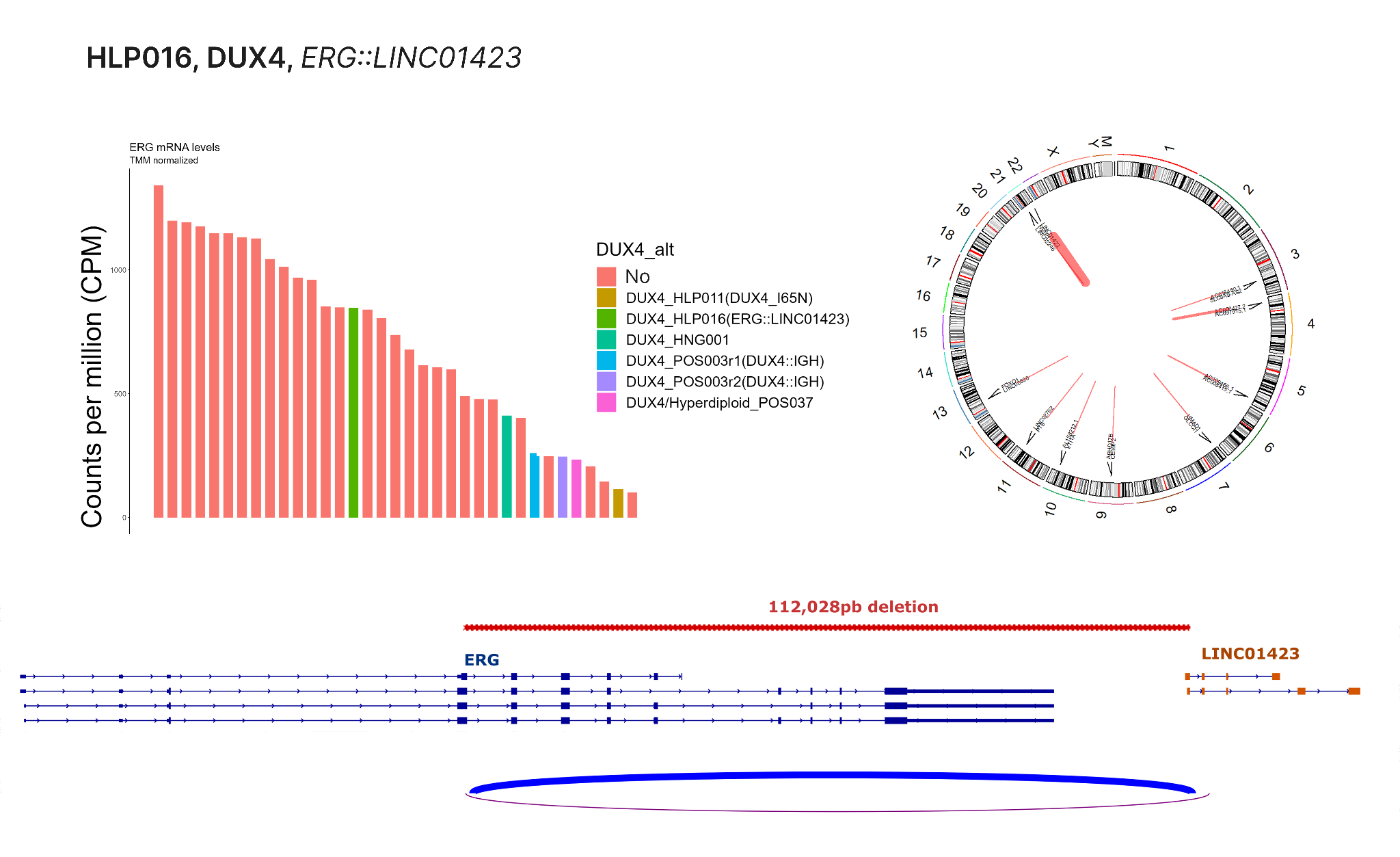
**

*4. Visualization of fusion transcripts identified by STAR-Fusion.*

Novel fusion genes were identified using STAR-Fusion (v1.11.0) and FusionInspector, ran on Singularity at the CCAD-UNC (Centro de Cómputo de Alto Desempeño, Universidad Nacional de Córdoba). Fusion transcripts were visualized per sample as circos plots, built using the *chimeraviz* package in R (Fig. S3).

*Supplementary Figure S3. Circos Plot for all patients evaluated in this study. Inter- or intrachromosomal fusion transcripts are represented in blue or red lines, respectively, and line thickness is proportional to the number of reads supporting the fusion transcript.*


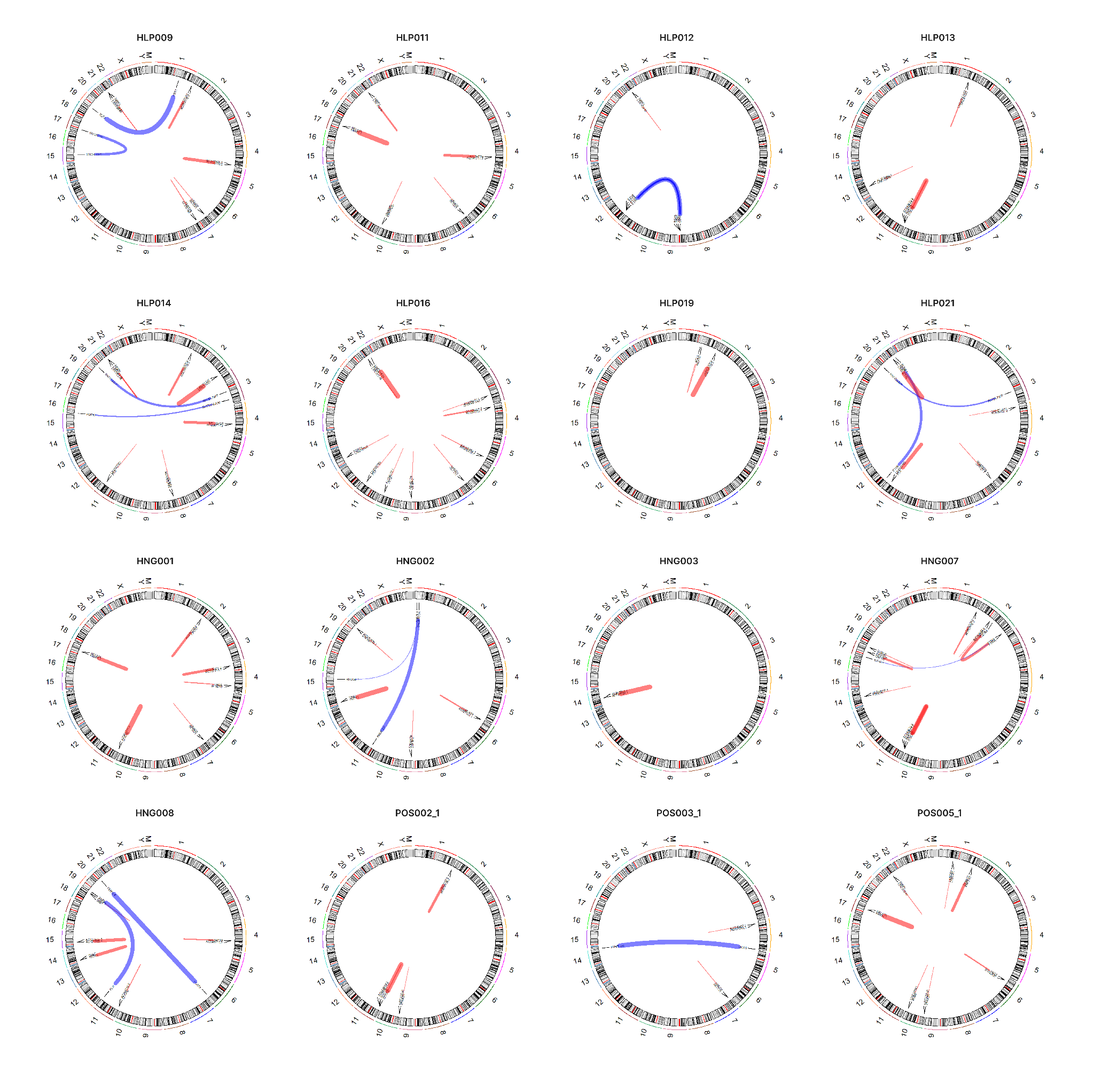


*Supplementary Figure S3 (cont).*


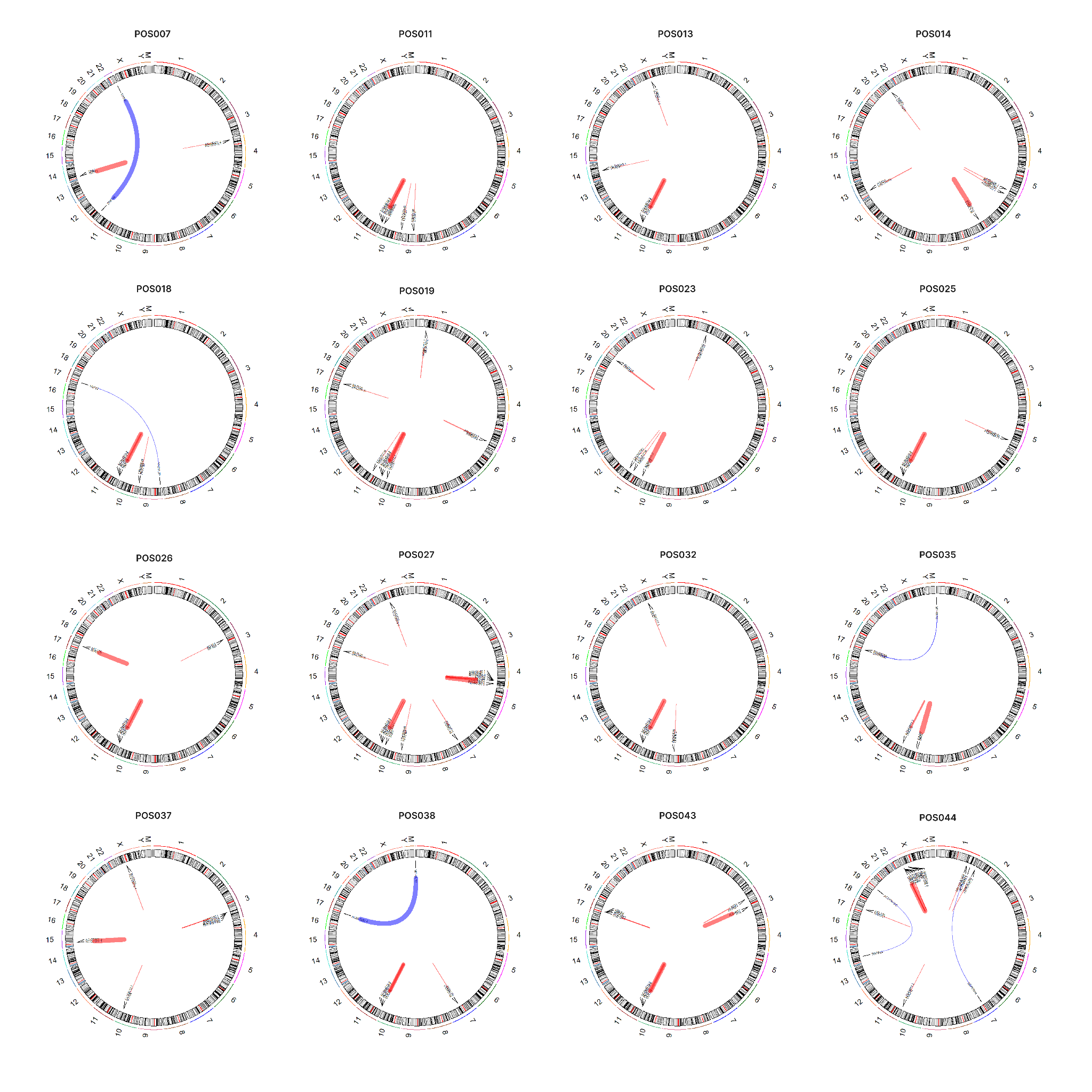


5. Evaluation of evolutionary conservation of protein residues corresponding to the novel variants identified in this study (Fig. S4).

*Supplementary Figure S4.* Logo Plots of *a) CREBBP, b) DUX4 and c) CSF3R were obtained using the VarSite tool. The positions involved in the nucleotide change are indicated by a square box.*

1. CREBBP p.G1542V. The Gly residue at position 1542 is very highly conserved (conservation = 1.0 from 185 aligned protein sequences).


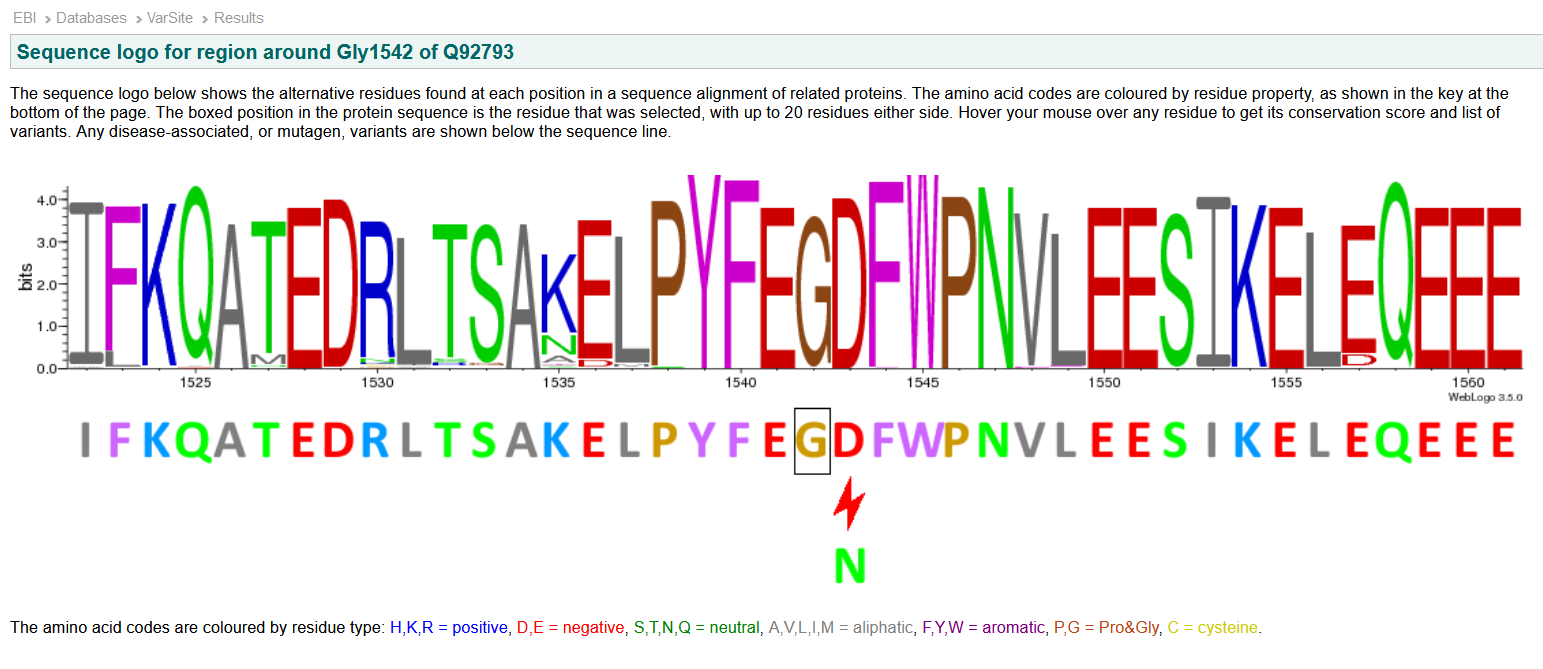


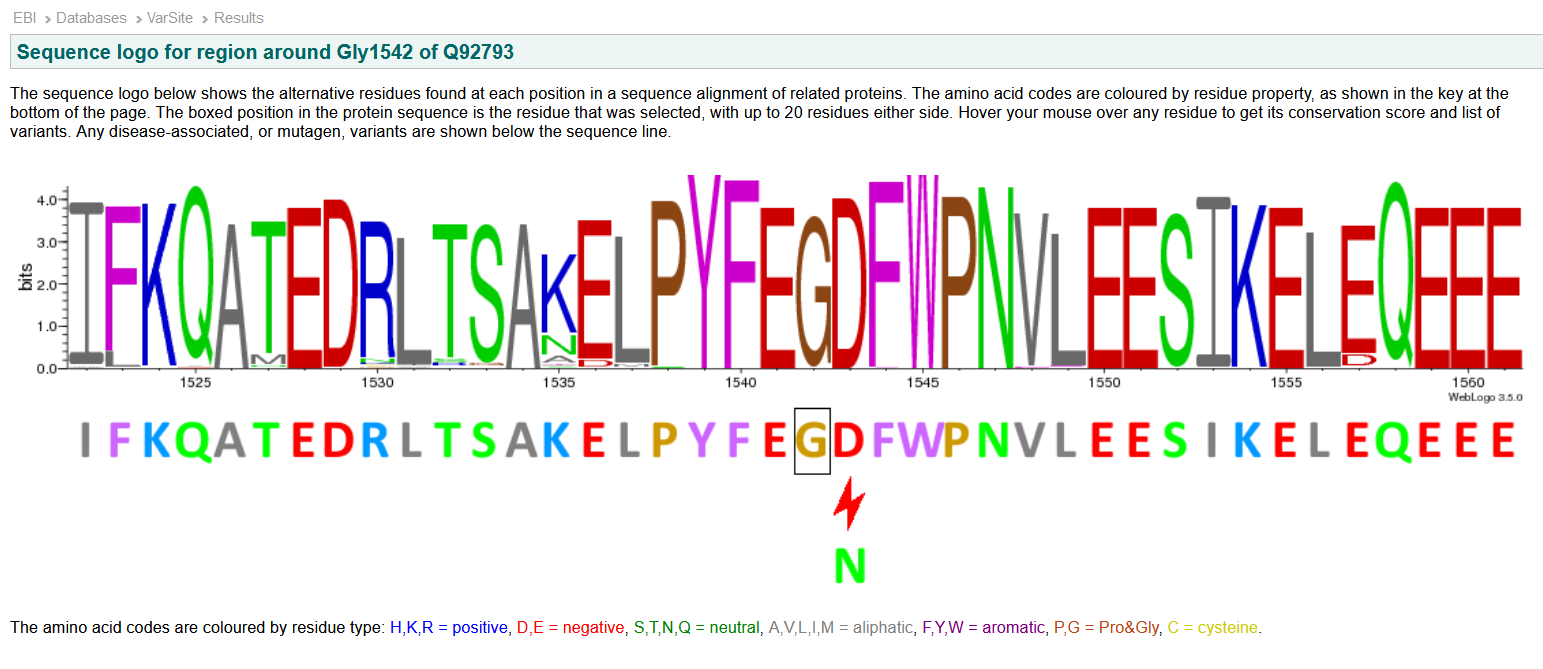


1. DUX4 p.I65N. The Ile residue at position 65 is very highly conserved (conservation = 1.0 from 30 aligned protein sequences).


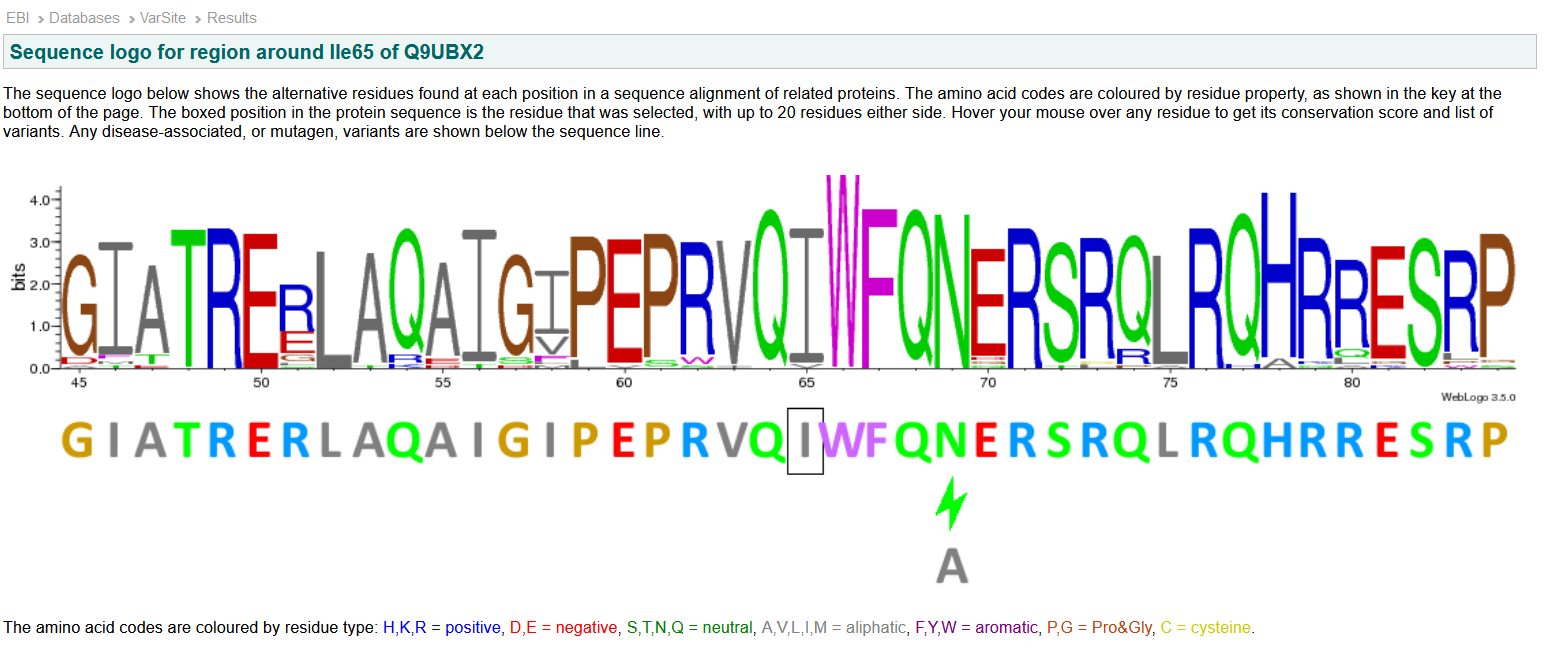


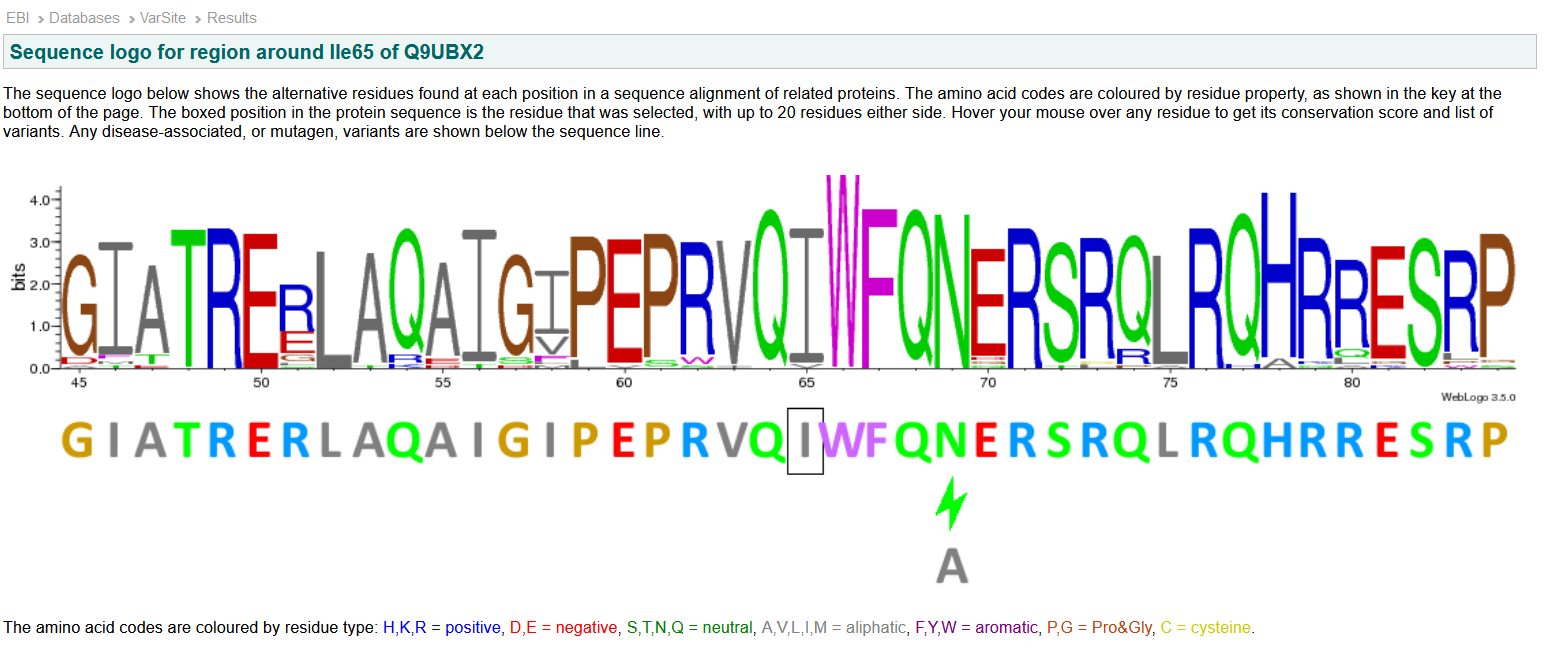


1. CSF3R p.G147R. The Gly residue at position 147 is highly conserved (conservation = 0.8 from 87 aligned protein sequences).


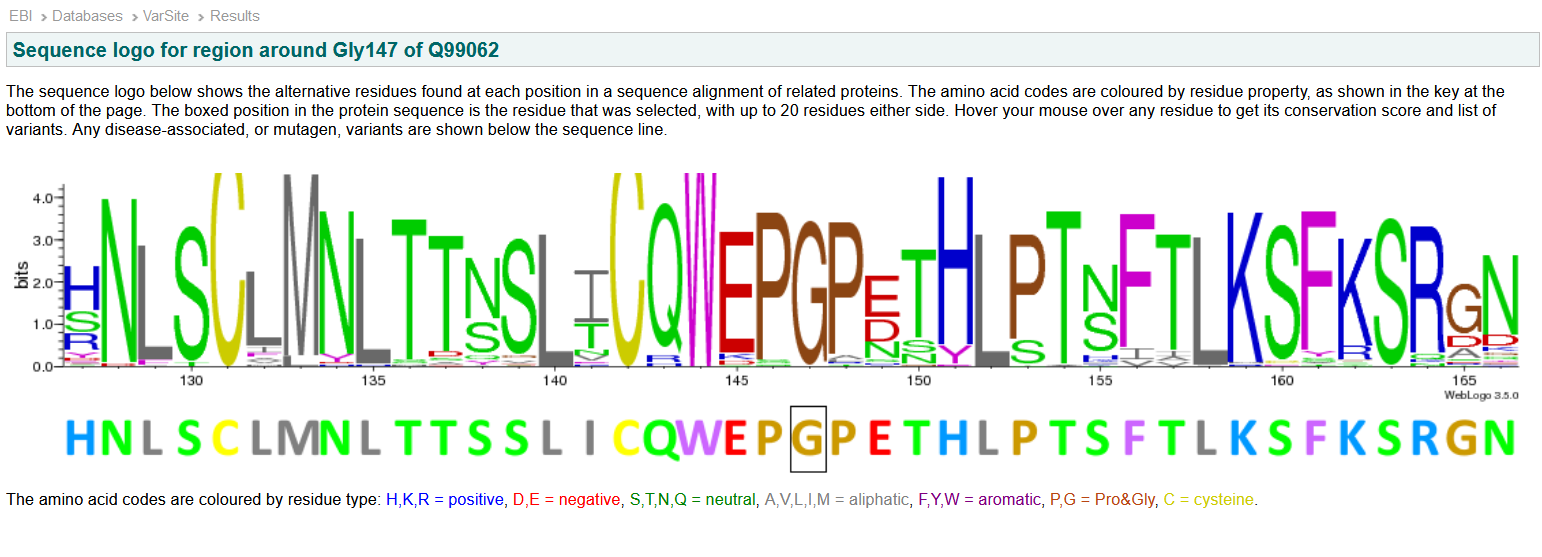

Supplement: Supplementary file 2 [file Supplementaryfile2.docx]
